# Antidepressant-Like Activity of Myelophil *via* Attenuation of Microglial-Mediated Neuroinflammation in Mice Undergoing Unpredictable Chronic Mild Stress

**DOI:** 10.3389/fphar.2019.00683

**Published:** 2019-06-13

**Authors:** Jin-Seok Lee, Won-Young Kim, Yoo-Jin Jeon, Sung-Bae Lee, Dong-Soo Lee, Chang-Gue Son

**Affiliations:** ^1^Institute of Traditional Medicine and Bioscience, Dunsan Hospital of Daejeon University, Daejeon, South Korea; ^2^Department of Internal Medicine, Daejeon St. Mary’s Hospital, The Catholic University of Korea, Daejeon, South Korea

**Keywords:** myelophil, depression, anxiety, antidepressants, microglia, serotonin

## Abstract

Myelophil, a 30% ethanol extract that has an equal rate in both *Astragali Radix* and *Salviae Radix*, is a remedy for the treatment of fatigue-linked disorders in traditional Oriental medicine. The majority of patients with chronic fatigue have a risk of comorbidity with depression symptoms. To evaluate the anti-depressant activity of Myelophil, mice were subjected to unpredictable chronic mild stress (UCMS, eight different stresses) for 3 weeks with daily administration of distilled water, Myelophil (25, 50, or 100 mg/kg), or *n*-acetyl-*l*-cysteine (NAC) (100 mg/kg). After the final stress exposure, three behavioral tests, including the open field test (OFT), forced swimming test (FST), and tail suspension test (TST), and stress-derived alterations of the serotonergic signal and inflammatory response in the hippocampus were measured. UCMS notably induced depressive behaviors, whereas these behavioral alterations were significantly reversed by the administration of Myelophil in regard to the OFT, FST, and TST results. Myelophil also significantly attenuated the over-activation of microglial cells and the inflammatory response in the hippocampal region (TNF-α, tumor necrosis factor-alpha; IL-1β, interleukin-1beta; and caspase-1). Furthermore, Myelophil significantly restored the distortions of serotonergic function in the dorsal raphe nuclei and neurogenesis in the subgranular zone of the hippocampus. These results support the clinical relevance of the anti-depressant activity of Myelophil, specifically by modulating serotonergic function and the neuroinflammatory response.

## Introduction

Depression, a pervasive emotional disorder, is the single largest contributor to the global burden of disease (WHO, 2017). The number of patients who suffer from depressive disorder is estimated to be 322 million worldwide (WHO, 2017). Its major symptoms, such as low mood, a feeling of sadness, and a loss of interest in things, lead to low socio-economic activity (Nestler et al., 2002). Annually, 0.8 million patients with depressive disorder commit suicide (Klonsky et al., 2016). However, the pathophysiological mechanisms have been unclear to date, and no curative therapeutics exists yet.

As a credible etiology, the hypothalamic–pituitary–adrenal (HPA) axis hypothesis is suggested (Pariante and Lightman, 2008). Accumulating evidence suggests that HPA axis hyperactivity induces the overproduction of brain pro-inflammatory cytokines, such as tumor necrosis factor-alpha (TNF-α) and interleukin-1beta (IL-1β), through microglial activation (Brites and Fernandes, 2015; Yirmiya et al., 2015), and this phenomenon has consistently been observed in subjects with depressive disorders (Zou et al., 2018). Recently, the role of Nucleotide-binding oligomerization domain (NACHT), Leucine-rich repeat (LRR), and Pyrin domation (PYD) domains-containing protein 3 (NLRP3) inflammasome-dependent IL-1β has emerged as a novel contributor to depressive disorders (Alcocer-Gomez and Cordero, 2014; Kaufmann et al., 2017). These molecular alterations eventually led to impaired serotonergic synthesis and neurotransmission, which are characteristics of depression (Velasquez and Rappaport, 2016). Therefore, maintenance of serotonergic homeostasis is considered as a main strategy for psychiatric disorders (Nordquist and Oreland, 2010).

Antidepressants have been developed on the basis of serotoninergic signal modulation, while selective serotonin reuptake inhibitors (SSRIs) and serotonin–norepinephrine reuptake inhibitors (SNRIs) represent the majority of the antidepressant market (Artigas, 2013). The global antidepressant market was estimated to be 11.6 billion dollars in 2017 (Mordor-Intelligence, 2017), and serotoninergic modulators account for 90% of market (Artigas, 2013). Nevertheless, current antidepressants have critical limitations, such as extensive adverse effects, poor treatment compliance and remission rate, and a high risk of relapse following drug withdrawal, for example, 61.8% in the case of fluoxetine (Gaynes et al., 2009; Andrews et al., 2012; Berwian et al., 2017).

Medicinal herbs have been attractive as candidates in antidepressant drug development (Lee and Bae, 2017). Myelophil, a 30% ethanol extract of *Astragali Radix* and *Salviae Radix*, is used to treat fatigue-associated disorders, including idiopathic chronic fatigue and chronic fatigue syndrome, in clinics of traditional Korean medicine. We previously presented that Myelophil exerted anti-fatigue in both clinical and experimental studies (Cho et al., 2009; Lee et al., 2015). Besides, our previous findings showed the neuropharmacological actions of Myelophil against hippocampal memory dysfunction, brain oxidative damage, and endocrine abnormality of HPA axis (Kim et al., 2013; Kim et al., 2014; Lee et al., 2014). However, there is no scientific evidence for the pharmacological property of Myelophil in depressive disorder. We hypothesized that Myelophil might have antidepressant-like effects because of the high relevance and comorbidity between chronic fatigue and depression (Demyttenaere et al., 2005; Penner and Paul, 2017).

The present study aimed to investigate the antidepressant-like properties of Myelophil and its underlying mechanisms using an unpredictable chronic mild stress (UCMS)-induced depression mouse model. In order to compare the relative pharmacological potential, NAC was adapted as a positive control.

## Materials and Methods

### Myelophil Preparation and Standardization

Myelophil is composed of 30% ethanol extract in equal amounts of *Astragali Radix* (*Astragalus membranaceus*) and *Salviae Radix* (*Salvia miltiorrhizae*). Myelophil was manufactured by Kyung-Bang Pharmacy (Incheon, Republic of Korea, lot. no. KB-Myelo-1801) according to the approved good manufacturing practice (GMP) guidelines of the Korean Ministry of Food and Drug Safety (MFDS). Fingerprinting analysis of Myelophil was performed to confirm the reproducibility as previously described (Lee et al., 2018). Briefly, ultra-high-performance liquid chromatography (UHPLC, Thermo Scientific, San Jose, CA, USA) coupled with a high-resolution LTQ Orbitrap mass spectrometry (MS) system (Thermo Scientific Co., San Jose, CA, USA) was used, and identifying analysis was conducted with each relative reference compound (rosmarinic acid; salvianolic acid A, B, C, and D; and formononetin).

### Animals and Stress Procedure

Forty-eight speciﬁc pathogen-free BALB/c male mice (8 weeks old, 22–24 g) were purchased from Dae Han Biolink Co., Ltd. (Eumseong, Republic of Korea). They were housed in plastic cages maintained at 23 ± 1°C with a 12-h light–dark cycle and freely fed food pellets (Cargill Agri Purina, Gyeonggido, Republic of Korea) and water. After acclimation for 1 week, the mice were randomly divided into six groups (*n* = 8): vehicle, UCMS, Myelophil (25, 50, or 100 mg/kg), and NAC (100 mg/kg, as a positive control) groups.

Animal care and experiments were conducted in accordance with the guidelines issued by the Institutional Animal Care and Use Committee of Daejeon University (Daejeon, Republic of Korea; Approval No. DJUARB 2017-017) and the Guide for the Care and Use of Laboratory Animals published by the United States National Institutes of Health.

The UCMS procedure was conducted as previously described (Nollet et al., 2013) with slight modifications. Briefly, except vehicle group, mice were subjected to a stress paradigm once per day over a period of 3 weeks: continuous illumination during the dark cycle, wet bedding for 24 h, isolation stress for 24 h, 45° tilting for 12 h, food and/or water deprivation for 12 h, restraint stress for 3 h, 4°C cold stress for 1 h, and swimming in cold water for 15 min. Detailed information for stress procedure is indicated in **Supplementary Figure 2**. After the final day of stress, the mice were sequentially subjected to behavioral tests to assess the depression/anxiety-like behavior [open field test (OFT) on day 22, a forced swimming test (FST) on day 23, and a tail suspension test (TST) on day 24]. During the entire experiment period, the mice were orally administered with distilled water (vehicle and UCMS), Myelophil, or NAC, respectively, at 11:00 am every day. The experimental scheme is summarized in **Supplementary Figure 2**.

### Open Field Test, Forced Swimming Test, and Tail Suspension Test

The OFT was performed as previously described (Ieraci and Herrera, 2006) with slight modification. The plastic enclosure box for the open field apparatus was contained in the black square side (40 × 40 × 30 cm), and the center of the field was distinguishable in the recording software. To evaluate the depressive and anxious conditions, each mouse was placed in the center of the field, and their spent time in the center zone and the total distance were subsequently recorded for 5 min at 25-lux illumination using a video camera connected to the corresponding software (Smart Junior).

The FST was performed as previously described (Porsolt et al., 1977; Chatterjee et al., 2012) with slight modification. The apparatus was contained in the plastic cylinder (30 × 30 × 50 cm), and it was filled with tap water at 25 ± 1°C up to 23 cm in height. Individual mouse was allowed to swim for 1 min (pre-test), and the immobility time (passive floating with no additional activity) and global activity (swimming duration) were recorded for 5 min at 30-lux illumination. The behavior between immobility and activity was judged by corresponding software (Smart Junior).

The TST was conducted as previously described (Steru et al., 1985; Chatterjee et al., 2012) with slight modification. The apparatus for the test consisted of a rectangular box (30 × 30 × 50 cm) with a rack on the top. Each mouse was individually suspended for 1 min for pre-test, and behaviors such as immobility and activity were recorded for 5 min at 25-lux illumination by software (Smart Junior).

Animal behavior tracking software (Smart Junior, Panlab SL; Barcelona, Spain) was used for recording distances, speeds, trajectories, and global activity (activity duration). Immobility and activity duration were judged by designated threshold (immobility, 0 to 120; low and high activities, 121 to 300). Behavioral tests were performed by researchers as a blind manner to experimental conditions.

### Sample Preparation

After the final behavioral tests, the mice were sacrificed under CO_2_ anesthesia on day 25. The serum was collected by centrifugation at 3,000 × *g* for 15 min. The brains of the five mice for each group were immediately removed and dissected to isolate the hippocampal tissue. The sera and hippocampi were stored at −80°C or RNAlater (Ambion, TX, USA) until use. The hippocampal tissue was homogenized in a radioimmunoprecipitation assay (RIPA) buffer, which was used for the biochemical analysis. For the immunohistological analysis, the remaining three mice for each group were subjected to transcardial perfusion with heparin (10 units/mL) and paraformaldehyde (PFA) solution, and the brains were maintained in 4% PFA solution. The total protein concentrations were measured using a bicinchoninic acid protein assay kit (Sigma). The absorbance at 560 nm was measured using a UV spectrophotometer (Molecular Devices Corp., Sunnyvale, CA, USA).

### Immunohistological Analysis of DCX, Iba-1, and 5-HT

The brains were gradually cryoprotected in 10%, 20%, and 30% sucrose for each 24-h interval and were subsequently embedded in tissue-freezing medium (Leica Microsystems, Bensheim, Germany) with liquid nitrogen. They were cut into frozen coronal sections (35 μm) using a Leica CM3050 cryostat. The sections were stored in free-floating buffer. For the immunohistological analysis, after washing with ice-cold PBS, the sections were blocked in 5% normal chicken serum (which contained 0.3% Triton X-100 in PBS) for 1 h. After being washed, the sections were incubated with primary antibodies against doublecortin (DCX, 1:200, sc-8066, Santa Cruz Biotechnology), ionized calcium binding adaptor molecule 1 (Iba-1, 1:200, 019-19741, Wako), or 5-hydroxytryptamine (5-HT, 1:200, ab66047, Abcam) overnight at 4°C. The sections were incubated with donkey anti-goat IgG H&L (1:400, Alexa Fluor^®^ 488, ab150129, Abcam) or goat anti-rabbit IgG Horseradish peroxidase (HRP) (1:400, ab6722, Abcam) secondary antibodies for 2 h at room temperature. For the Iba-1-positive signal, the sections were subsequently exposed to an avidin–biotin peroxidase complex (Vectastain ABC kit, Vector Laboratories) for 2 h. The peroxidase activity was visualized using a stable diaminobenzidine solution. For the DCX- and 5-HT-positive signal, the sections were subsequently exposed to 4', 6-Diamidino-2-Phenylindole, Dihydrochloride (DAPI) (1:1,000, D9542, Sigma) to stain cell nuclei. Immunoreactions were observed under an Axio-phot microscope (Carl Zeiss, Germany), and the signals were quantified using ImageJ 1.46 software (NIH, Bethesda, MD, USA).

### Determination of Corticosterone and Pro-Inflammatory Cytokines

The serum corticosterone level was determined using a DetectX^®^ corticosterone Arbor assay kit (Ann Arbor, MI, USA). The absorbance at 450 nm was measured using a UV spectrophotometer. The levels of pro-inflammatory cytokines in the hippocampal homogenates were determined using commercially available enzyme immunoassay (EIA) kits for TNF-α (BD Biosciences, San Diego, CA, USA) and IL-1β (R&D Systems Inc., Minneapolis, MN, USA), and the absorbance was read within 10 min at 450 and 570 nm using a UV spectrophotometer (Molecular Devices).

### Determination of Caspase-1 Activity

The caspase-1 activity in the hippocampal homogenates was measured using a mouse caspase-1 enzyme-linked immunosorbent assay kit (Novus Biologicals, Littleton, CO, USA). The absorbance at 450 nm was measured using a UV spectrophotometer (Molecular Devices).

### Western Blotting Analysis

The protein expressions of NACHT, LRR, and PYD Domains-Containing Protein 3 (NLRP3), apoptosis-associated Speck-like protein containing a CARD (ASC), pro-IL-1β and mature IL-1β, and β-actin in the hippocampal homogenates were evaluated using a western blotting method. The protein concentration of the homogenates was equalized, and the samples were separated by 10% polyacrylamide gel electrophoresis and transferred to polyvinylidene fluoride (PVDF) membranes. To minimize the non-specific binding, the membrane was blocked in 5% bovine serum albumin for 1 h. The membranes were incubated with primary antibodies, such as NLRP3 (1:500, ab214185, Abcam), ASC (1:500, sc-271054, Santa Cruz), pro-IL-1β and mature IL-1β (1:1,000, ab9722, Abcam), or β-actin (1:2500, PA1-183, Thermo-Fisher Scientific), overnight at 4°C. After being washed, the membranes were incubated with an HRP-conjugated anti-rabbit or anti-mouse antibody (GeneTex, Inc., Irvine, CA) for 1 h. The western blotting results were visualized with an enhanced chemiluminescence (ECL) advanced kit. The intensity was analyzed with ImageJ version 1.46 (NIH, Bethesda, MD, USA).

### Determination of Nitric Oxide in BV2 Microglial Cells

Mouse microglia cells (BV2 cell line) were cultured in DMEM supplemented with 10% Fetal Bovine Serum (FBS) and 1% penicillin–streptomycin. BV2 cells were incubated at 37°C under 5% CO_2_. The BV2 cells were seeded at 2 × 10^4^ cells/well into 96-well microplates. After incubation for 12 h, the BV2 cells were pre-treated with Myelophil (5, 10, or 20 μg/mL) or NAC (100 μM) for 2 h. After exposure to the gram-negative lipopolysaccharide bacteria (LPS, 1 μg/mL) for 24 h, the nitric oxide levels of the cell supernatants were determined using the previously described method (Green et al., 1982). Briefly, the cell supernatant was responded by the Griess reagent [1% sulfanilamide, 0.1% *N*-(1-naphthyl) ethylenediamine hydrochloride, and 2.5% H_3_PO_4_] at 37°C for 20 min, and the resulting purple azo dye product was subsequently measured at 540 nm using a UV spectrophotometer (Molecular Devices).

### Statistical Analysis

Data are expressed as the mean ± standard error of the mean (SEM). The statistically significant differences between the groups were evaluated by one-way analysis of variance (ANOVA) followed by *post hoc* multiple comparisons with Tukey’s honestly significant difference (HSD) test using IBM SPSS statistics software, ver. 25.0 (SPSS Inc., Chicago, IL, USA). Differences at *p* < 0.05 indicate statistical significance.

## Results

### Compounds Present in Myelophil

The major peaks and their retention times, formulae, and molecular weight were consistent with previous analysis (Lee et al., 2018). Six major peaks were detected at 7.47, 8.14, 8.27, 8.71, 9.38, and 12.65 min of retention time under the UV wavelength of 254 nm. Their mass to charge ratios (*m*/*z*) were analyzed in 419.0969 (salvianolic acid D), 361.0917 (rosmarinic acid), 493.1125 (salvianolic acid C), 719.1604 (salvianolic acid B), 495.1276 (salvianolic acid A), and 269.0806 (formononetin) *m*/*z* as displayed in **Supplementary Figure 1**.

### Effects on Depressive and Anxious-Like Behaviors

**Figure 1:**
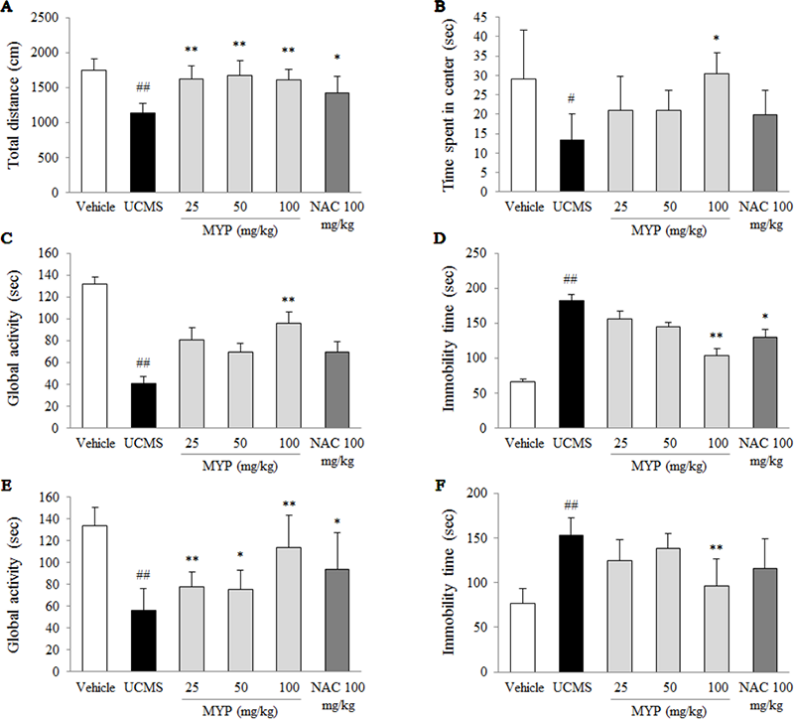
Depressive and anxiety behavioral tests. After unpredictable chronic mild stress (UCMS) with/without oral administration of Myelophil (MYP) or *n*-acetyl-*l*-cysteine (NAC) for 21 days, the total distance **(A)** and spent time in the center zone **(B)** in the open field test (on the 22nd day), the global activity **(C)** and immobility time **(D)** in the forced swimming test (on the 23rd day), and the global activity **(E)** and immobility time **(F)** in the tail suspension test (on the 24th day) were assessed. The data are expressed as the mean ± standard error of the mean (SEM) (*n* = 8). Significant differences were evaluated by one-way analysis of variance (ANOVA) with Tukey’s honestly significant difference (HSD) *post hoc* test. ^#^ *p* < 0.05 and ^##^ *p* < 0.01 compared with the vehicle-treated group; *p < 0.05 and **p < 0.01 compared with the UCMS-subjected group.

As an anxiety-based test, the OFT was conducted, and the UCMS significantly decreased the total distance [*F*(5, 42) = 12.68; *p* < 0.001] (*p* < 0.01) and time spent in the center zone [*F*(5, 42) = 5.69; *p* < 0.001] (*p* < 0.05) compared with those in the vehicle-treated group. These reductions were completely reversed by the treatment of Myelophil, particularly at the dose of 100 mg/kg, compared with those in the UCMS group (*p* < 0.05 and *p* < 0.01, respectively, **Figure 1A and B**).

To evaluate depressive-like behaviors, the forced swimming test (FST) and tail suspension test (TST) were performed. Regarding the FST results, the UCMS significantly reduced the global activity [*F*(5, 42) = 18.59; *p* < 0.001] and elevated immobility time [*F*(5, 42) = 19.96; *p* < 0.001] compared with those in the vehicle-treated group (*p* < 0.01 for both parameters). Myelophil treatment, however, showed noteworthy antidepressant-like activity particularly for the 100 mg/kg dose (*p* < 0.01, **Figure 1C and D**).

A low global activity [*F*(5, 42) = 42.55; *p* < 0.001] and prolonged immobility time [*F*(5, 42) = 55.84; *p* < 0.001] in the TST were induced by the UCMS, and they were significantly different compared with those in the vehicle-treated group (*p* < 0.01 for both parameters). Myelophil treatment (100 mg/kg) significantly attenuated these behavioral alterations compared with those in the UCMS group (*p* < 0.01, **Figure 1E and F**). NAC also showed positive effects in the depressive and anxious-like behaviors compared with those in the vehicle-treated group; however, these effects were not significant in all tests.

### Effects on Microglial Activation in Hippocampus

**Figure 2:**
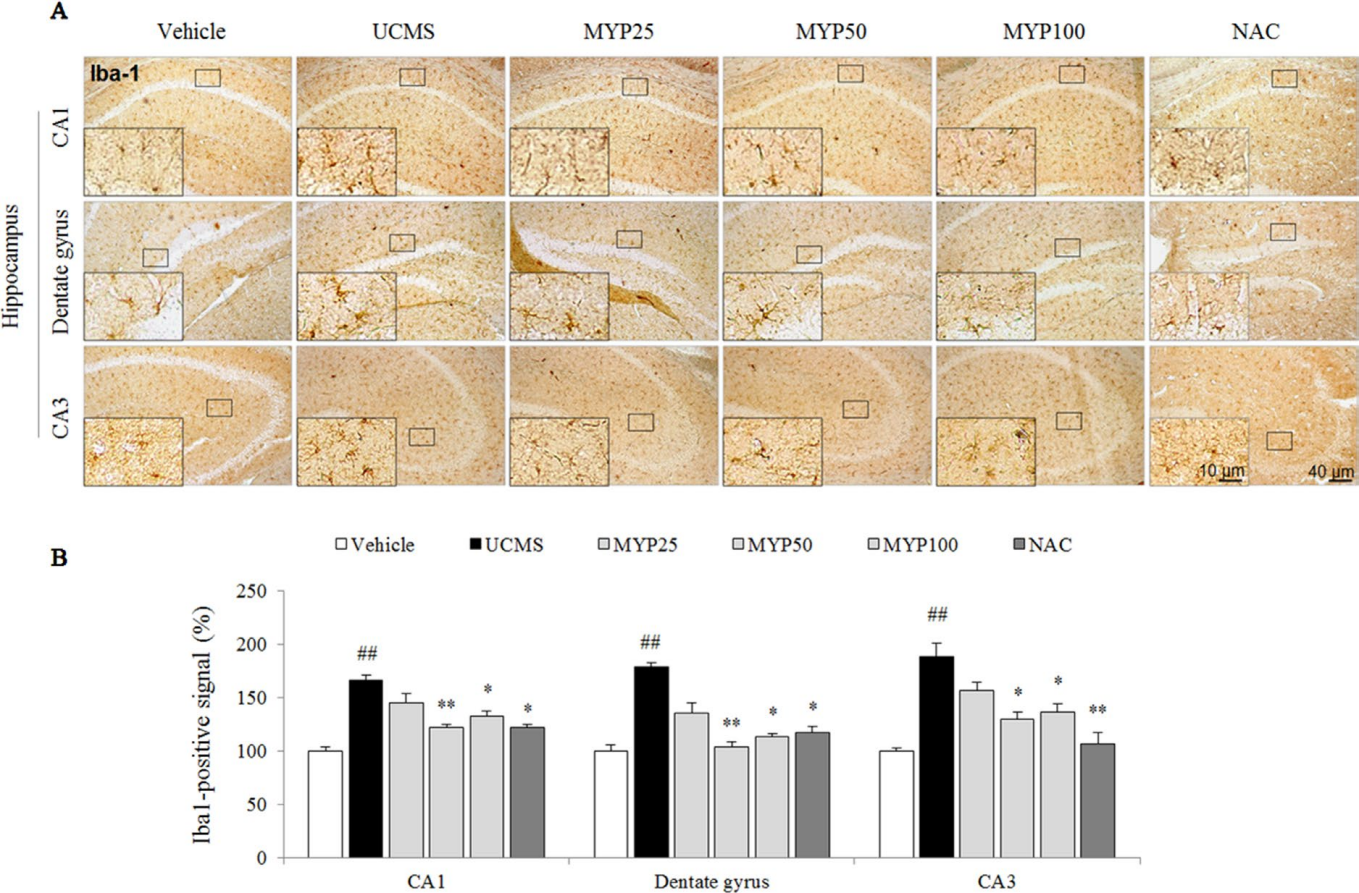
Microglial activation in CA1, dentate gyrus, and CA3 of hippocampus. After behavioral tests, mice were sacrificed on the 25th day. Microglial activation was evaluated with Iba-1 immunofluorescence analysis in the hippocampus CA1, dentate gyrus, and CA3 region **(A)**, and its signal was semi-quantified **(B)**. The data are expressed as the mean ± SEM (*n* = 3). Significant differences were evaluated by one-way ANOVA with Tukey’s HSD *post hoc* test. ^##^ *p* < 0.01 compared with the vehicle-treated group; *p < 0.05 and **p < 0.01 compared with the UCMS-subjected group.

The UCMS remarkably increased the microglial activation in cornus ammonis (CA)1 [*F*(5, 12) = 20.52; *p* < 0.001], dentate gyrus [*F*(5, 12) = 23.88; *p* < 0.001], and CA3 region [*F*(5, 12) = 14.28; *p* < 0.001] of the hippocampus (*p* < 0.01 for all regions). Hyper-activation of microglia cell was significantly attenuated by the Myelophil treatment especially in doses of 50 and 100 mg/kg (*p* < 0.05 or *p* < 0.01, **Figure 2A and B**). NAC also showed similar effects against microglial activation.

### Effects on Serum Corticosterone

**Figure 3:**
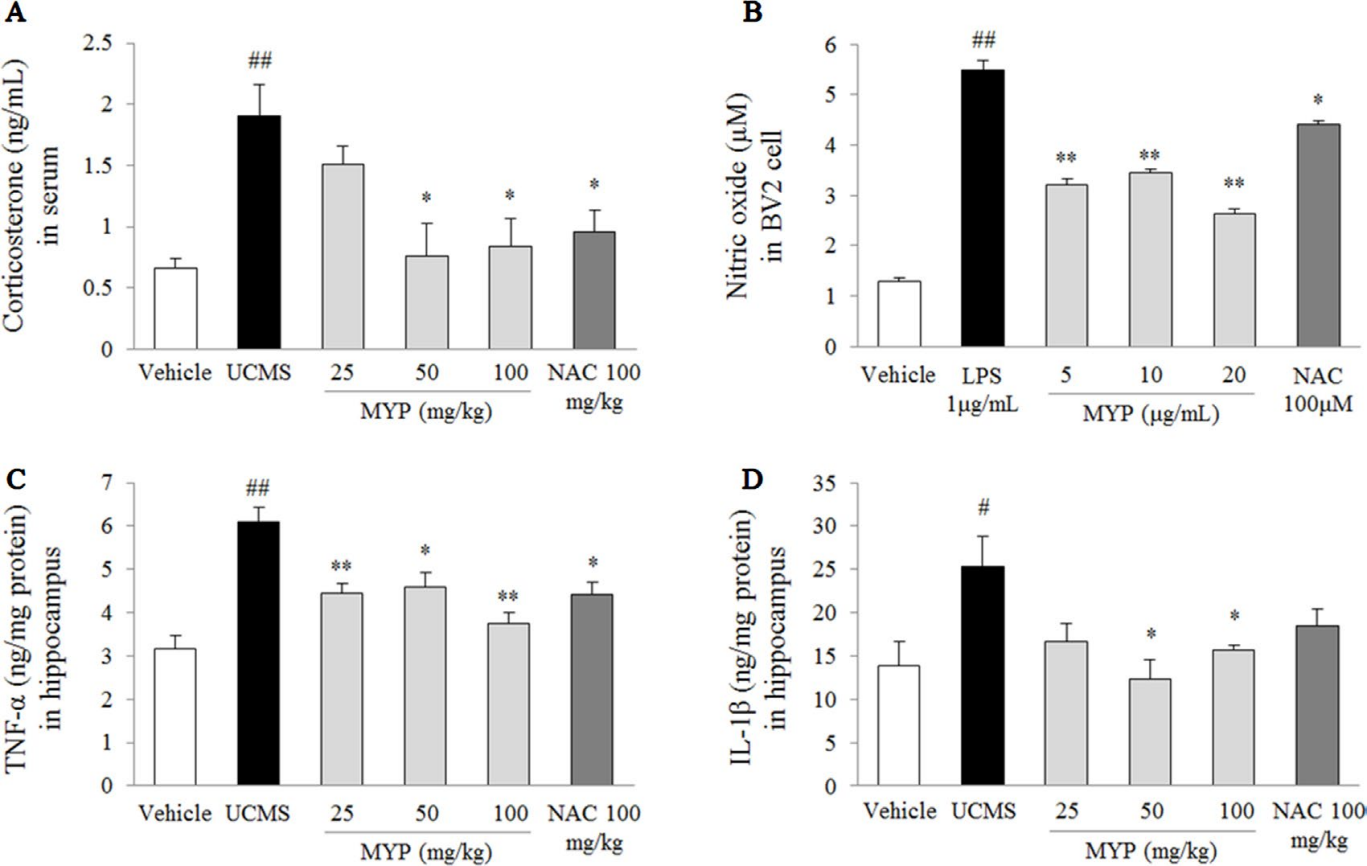
Serum corticosterone and nitric oxide in BV2 cells and pro-inflammatory cytokines in hippocampus. After behavioral tests, mice were sacrificed on the 25th day. Serum corticosterone was evaluated *via* enzyme immunoassay (EIA) method **(A)**, nitric oxide in BV2 microglial cells was evaluated by Griess method **(B)**, and hippocampal tumor necrosis factor (TNF)-α **(C)** and interleukin (IL)-1β **(D)** were measured by EIA method. The data are expressed as the mean ± SEM (*n* = 5). Significant differences were evaluated by one-way ANOVA with Tukey’s HSD *post hoc* test. ^#^ *p* < 0.05 and ^##^ *p* < 0.01 compared with the vehicle-treated group or cells; *p < 0.05 and **p < 0.01 compared with the UCMS-subjected group or lipopolysaccharide-treated cells.

The UCMS significantly increased the serum corticosterone (2.9-fold) compared with that in the vehicle-treated group [*F*(5, 24) = 4.69; *p* = 0.004] (*p* < 0.01), while the Myelophil-treated group showed significant reductions for the high corticosterone level (*p* < 0.05 for both 50 and 100 mg/kg, **Figure 3A**). Treatment with NAC also showed similar effects as Myelophil.

### Effects on Nitric Oxide in BV2 Microglia Cells

The LPS induced an inflammatory response in BV2 cells, as evidenced by the increase of nitric oxide [*F*(5, 24) = 99.67; *p* < 0.001] (4.3-fold, *p* < 0.01) in the cell supernatant. Pretreatment with Myelophil inhibited the highly increased nitric oxide level than did the LPS-treated cells (*p* < 0.01 for all doses, **Figure 3B**). Nitric oxide scavenging activity was also present in NAC-pretreated cells similar to Myelophil.

### Effects on Pro-Inflammatory Cytokines in Hippocampus

The UCMS-subjected group showed significant elevations of TNF-α [*F*(5, 24) = 9.29; *p* < 0.001] (1.9-fold) and IL-1β [*F*(5, 24) = 2.67; *p* = 0.047] (1.8-fold) in the hippocampal tissue than did the vehicle-treated group (*p* < 0.05 and *p* < 0.01, respectively). These elevations were significantly attenuated by Myelophil treatment compared with the UCMS group: TNF-α level (*p* < 0.05 for 25 and 100 mg/kg, *p* < 0.01 for 50 mg/kg) and IL-1β level (*p* < 0.05 for 50 and 100 mg/kg; **Figure 3C and D**). The anti-inflammatory effects of NAC were only present in the TNF-α result.

### Effects on Hippocampal NLRP3 Inflammasome

**Figure 4:**
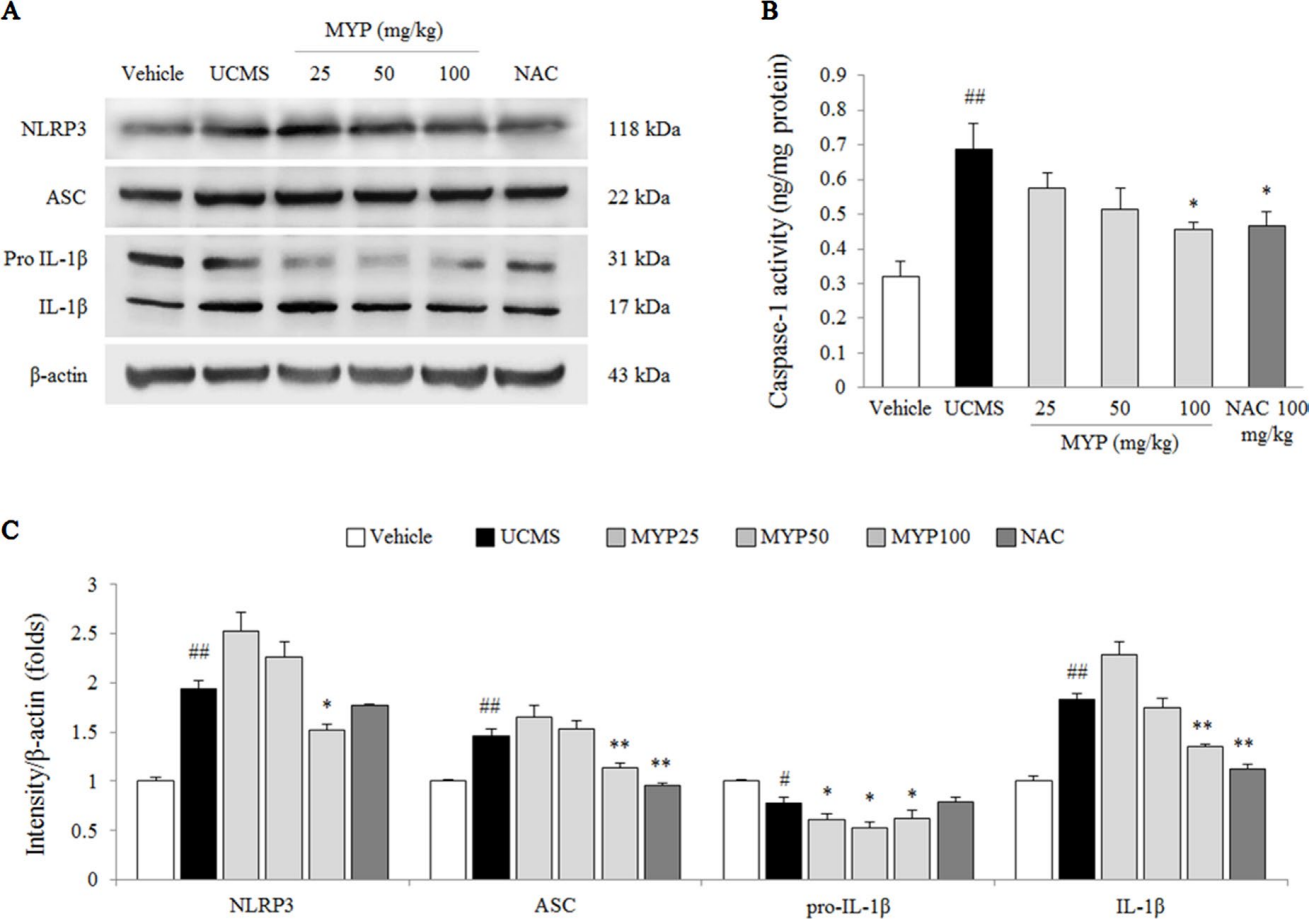
NLRP3 inflammasome in hippocampus. After behavioral tests, mice were sacrificed on the 25th day. Protein levels of NACHT, LRR, and PYD domains-containing protein (NLRP) 3, apoptosis-associated Speck-like protein containing a CARD (ASC), and pro-IL-1β and mature IL-1β **(A)** were determined by western blotting method. Caspase-1 activity in the hippocampus was measured by EIA method. Each protein expression was semi-quantified **(C)**. The data are expressed as the mean ± SEM (*n* = 5). Significant differences were evaluated by one-way ANOVA with Tukey’s HSD *post hoc* test. ^#^ *p* < 0.05 and ^##^ *p* < 0.01 compared with the vehicle-treated group; *p < 0.05 and **p < 0.01 compared with the UCMS-subjected group.

The UCMS significantly activated the NLRP3 inflammasome, as evidenced by NLRP3 [*F*(5, 24) = 143.56; *p* < 0.001] (2-fold, *p* < 0.01), ASC [*F*(5, 24) = 113.80; *p* < 0.001] (1.5-fold, *p* < 0.01), pro-IL-1β [*F*(5, 24) = 50.25; *p* < 0.001] (*p* < 0.05), and mature IL-1β [*F*(5, 24) = 220.91; *p* < 0.001] (1.9-fold, *p* < 0.01) compared with those in the vehicle-treated group. Myelophil treatment notably inhibited against NLRP3 inflammasome activation; the differences in NLRP3, ASC, pro-IL-1β, and mature IL-1β were statistically significant compared with those in the UCMS group (*p* < 0.05 or *p* < 0.01 for 100 mg/kg, **Figure 4A and C**).

### Effects on Caspase-1 Activity in Hippocampus

The caspase-1 activity in the hippocampal tissue of the UCMS-subjected group was higher than that in the vehicle-treated group [*F*(5, 24) = 5.17; *p* = 0.002] (approximately 2.1-fold, *p* < 0.01), whereas the hyper-activity of caspase-1 was significantly inhibited by Myelophil treatment compared with the UCMS group (*p* < 0.05 for 100 mg/kg, **Figure 4B**). NAC treatment also had a similar effect on the caspase-1 hyper-activation.

### Effects on Hippocampal Neurogenesis and Serotonergic Function in Dorsal Raphe Nuclei

**Figure 5:**
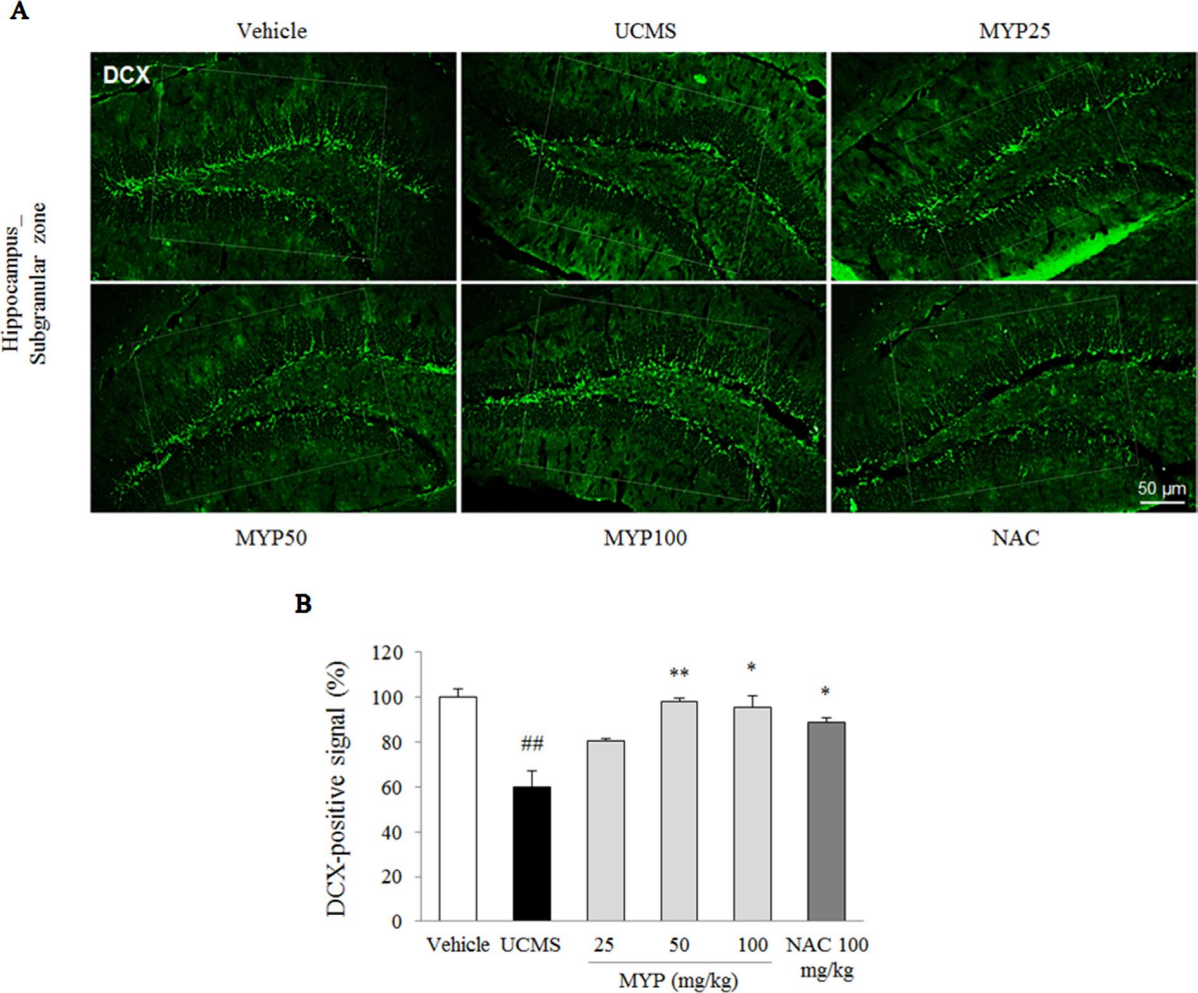
Hippocampal neurogenesis. After behavioral tests, mice were sacrificed on the 25th day. Hippocampal neurogenesis was confirmed by doublecortin (DCX) immunofluorescence analysis in the hippocampus dentate gyrus regions **(A)**, and its signal was semi-quantified **(B)**. The data are expressed as the mean ± SEM (*n* = 3). Significant differences were evaluated by one-way ANOVA with Tukey’s HSD *post hoc* test. ^##^ *p* < 0.01 compared with the vehicle-treated group; *p < 0.05 compared with the UCMS-subjected group.

**Figure 6:**
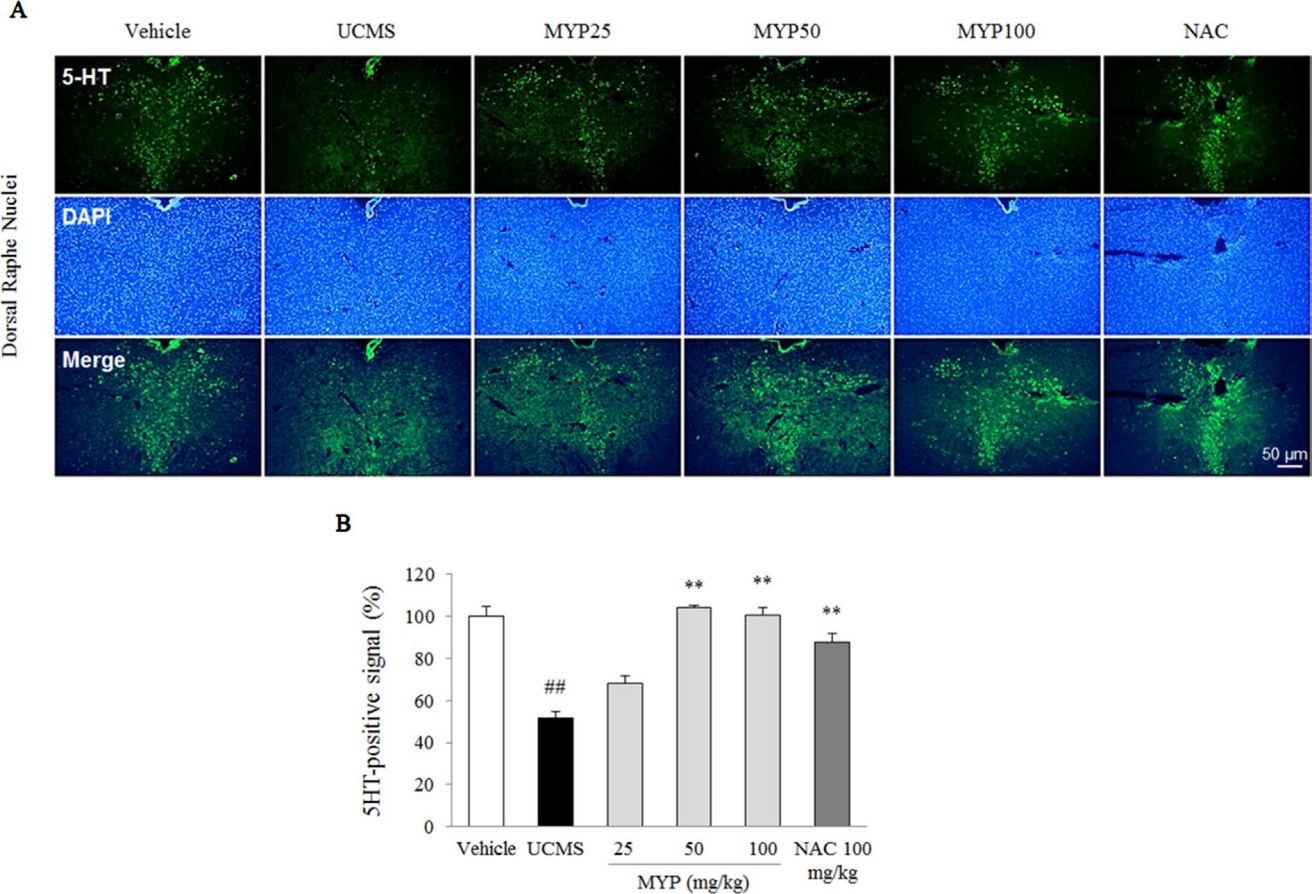
Serotonergic signal in dorsal raphe nuclei. After behavioral tests, mice were sacrificed on the 25th day. Serotonergic signal was confirmed by 5-hydroxytryptamine (5-HT) immunofluorescence analysis in the dorsal raphe nuclei **(A)**, and its signal was semi-quantified **(B)**. The data are expressed as the mean ± SEM (*n* = 3). Significant differences were evaluated by one-way ANOVA with Tukey’s HSD *post hoc* test. ^##^ *p* < 0.01 compared with the vehicle-treated group; **p < 0.01 compared with the UCMS-subjected group.

The UCMS exerted a predominant reduction of the 5-HT activity in the region of the dorsal raphe nuclei compared with those in the vehicle-treated group [*F*(5, 12) = 33.42; *p* < 0.001] (*p* < 0.01), whereas treatment with Myelophil notably recovered the low 5-HT activity (*p* < 0.01 for both 50 and 100 mg/kg). The DCX-positive signal in the subgranular zone of the hippocampus was markedly decreased by UCMS compared with that in the vehicle-treated group [*F*(5, 12) = 14.13; *p* < 0.001] (*p* < 0.01). Moreover, an increase of DCX-positive neuronal dendrites was observed in the Myelophil-treated group (*p* < 0.01 for 50 mg/kg, *p* < 0.05 for 100 mg/kg). NAC also showed similar effects as Myelophil. The immunofluorescence staining results were quantified, and there was a significant difference between the UCMS-subjected and Myelophil-treated groups (*p* < 0.01, **Figures 5** and **6**).

## Discussion

In present study, we found that Myelophil could alleviate the depressive-like behaviors *via* modulation of microglial-mediated neuroinflammation. Our findings are the first evidence of the antidepressant-like effects of Myelophil, which indicates the therapeutic possibilities on the mood disorders.

To verify the hypothesis that Myelophil exerts antidepressant-like effect, we chose the BALB/c mouse strain to induce depressive behaviors because it is known to be more susceptible to UCMS relative to other rodent strains (Farley et al., 2012), and the UCMS method has been commonly used as a depressive and anxious animal model (Nollet et al., 2013). The reliability of the UCMS animal model for inducing depression has been validated by numerous studies, and the unpredictable stress model can mimic the human psychopathology (Willner, 2017). We also adopted the FST, TST, and OFT as the behavioral tests, which are representative assessments for evaluating antidepressant interventions (Cryan et al., 2005; Slattery and Cryan, 2012; Daniel et al., 2017). As expected, chronic exposure to unpredictable stress led to predominant behavioral alterations in the FST, TST, and OFT, while these depression-related behaviors were significantly ameliorated by administration of Myelophil (**Figure 1A to F**). These Myelophil-derived results were similar with those of another study using hesperidin (a kind of citrus bioflavonoid) under UCMS-induced depressive behavioral tests (Fu et al., 2019).

To explain the underlying mechanisms of Myelophil, we subsequently examined the stress-responsive system, particularly focusing on the HPA axis hyper-activation. The HPA axis abnormality has been implicated in the pathophysiology of major depressive disorder. Hypercortisolemia is commonly observed in 40% to 60% of depressed patients (Murphy, 1991). The glucocorticoid receptor-mediated negative regulation of cortisol release is impaired in conditions of psychiatric pathology (Pariante and Lightman, 2008). In a previous animal study, chronically dexamethasone-injected mice have shown depressive-like behaviors and a decrease of hippocampal gene expression for glucocorticoid receptor (Skupio et al., 2015). Consistently with previous reports, we verified the increase of serum corticosterone by UCMS. However, this over-release of corticosterone was significantly attenuated by Myelophil administration, which proposed the antidepressant-like activity of Myelophil *via* balancing the endogenous glucocorticoid system (**Figure 3A**).

High glucocorticoids lead to morphological and functional changes of microglial cells in the brain, for example, from resting state into reactive phenotype as a neuroinflammatory response (Nair and Bonneau, 2006). Microglia, resident immune cells in the central nervous system, plays a pivotal role in the pathogenesis of depression (Yirmiya et al., 2015). Antidepressants, such as imipramine, suppressed the M1 phenotype microglia in the hippocampus of mice exposed to chronic mild stress (Zhao et al., 2016). As expected, the administration of Myelophil predominantly attenuated the over-activation of microglia in the CA1, dentate gyrus, and CA3 of the hippocampus (**Figure 2A and B**). Microglial activation in the hippocampal granule cell layer, hilus, CA1, and CA3 regions was known to trigger the onset of depression (Iwata et al., 2016). We further confirmed the inhibitory effects of Myelophil against the LPS-induced production of nitric oxide in the BV2 murine microglia cell line (**Figure 3B**). Researchers in pharmacology are attempting to identify the antidepressant agents that involve the regulation of microglia-derived neuroinflammation (Chen et al., 2017). In our study, the hippocampal pro-inflammatory cytokines, including TNF-α and IL-1β, were also completely normalized by Myelophil treatment (**Figure 3C and D**).

Emerging evidence reinforces the importance of NLRP3 inflammasome in neuropsychiatric disorders, particularly in depression and anxiety (Velasquez and Rappaport, 2016; Song et al., 2017). One preclinical study showed that 4 weeks of UCMS in mice resulted in depressive-like behaviors, and its contributing factor was NLRP3 inflammasome in the hippocampal region (Zhang et al., 2015). As expected, we found that Myelophil (100 mg/kg) significantly inhibited the hippocampal protein expressions of NLRP3, ASC, and caspase-1 activity (**Figure 4A–C**). These results implied the possibility of an NLRP3-dependent antidepressant-like action of Myelophil. In a case–control study, 20 patients with major depressive disorder exhibited high anxiety score and increased levels of caspase-1 and NLRP3 expression in peripheral blood mononuclear cells than did the healthy group (Alcocer-Gomez et al., 2016). The glucocorticoid-induced NLRP3 inflammasome formation was observed in a primary microglia cell isolated from mouse hippocampus (Frank et al., 2014). Furthermore, one clinical study identified a high correlation between the glial activation level and depressive score in patients with CFS using positron emission tomography (PET) (Nakatomi et al., 2014). These facts support that antidepressant-like effects of Myelophil are linked to anti-fatigue activity.

Excessive microglial activation causes an impairment of the hippocampal neurogenesis under conditions of stress and inflammation (Sierra et al., 2014). In addition, the reduced hippocampal neurogenesis is closely involved in the pathophysiology of major depression (Snyder et al., 2011). Our data also showed a notable suppression of the hippocampal neurogenesis, which was significantly ameliorated by Myelophil treatment (**Figure 5**). It is known that chronic antidepressant treatment (serotonergic modulator) increases adult hippocampal neurogenesis in both non-human primates and humans (Perera et al., 2011; Boldrini et al., 2012). Serotonin, 5-HT, as a monoamine neurotransmitter, is the most important target molecule in depressive disorder. Therefore, serotonin-target medications, such as SSRIs and SNRIs, are prescribed most frequently in approximately 90% of cases (Artigas, 2013). It is particularly interesting that dysregulation of the HPA axis attenuates 5-HT neurotransmission (Mahar et al., 2014). In our results, the altered 5-HT signals in the dorsal raphe nuclei were restored by Myelophil treatment (**Figure 6A and B**).

Myelophil was developed for the treatment of fatigue-related disorders on the basis of the theory of traditional Chinese/Korean medicine. *Astragali Radix* and *Salviae miltiorrhizae Radix* are the main materials in Myelophil, and these two herbal plants are commonly used to maintain qi and bloodstream homeostasis in the human body (Cho et al., 2009). From our previous studies, Myelophil showed pharmacological actions against fatigue-related pathology such as brain oxidative stress and memory deficit (Lee et al., 2012; Lee et al., 2015). Our present data supported the applicability of Myelophil to depressive symptoms, which clearly accompany chronic fatigue disorders. However, the present study has a limitation of unknown information regarding the active compounds that correspond to the anti-depressive property. A study found that salvianolic acid B, a major compound in *Salviae Radix*, exhibits anti-inflammatory effects by modulating NLRP3 inflammasome (Jiang et al., 2017). Salvianolic acid B promotes microglial M2-polarization against UCMS-induced M1 phenotype (Zhang et al., 2017). Astragaloside IV, a main *Astragali Radix* compound, inhibits depressive-like behaviors by regulating the nuclear factor-κB/NLRP3 axis (Song et al., 2018). Further studies are required to identify the major active compound in the future. We used NAC as a positive control because the antidepressant-like effects of NAC have been explored in several studies (Ferreira et al., 2008; Costa-Campos et al., 2013). One study group is planning to evaluate the inflammation-inhibiting NAC effects on depressive disorder in a randomized placebo-controlled trial (Yang et al., 2018).

Taken together, our findings comprise the first evidence for the antidepressant-like effects of Myelophil, and its underlying mechanism may involve the regulation of NLRP3-dependent neuroinflammation and the serotonergic signal. This study would support the pharmacological applicability of Myelophil in chronic fatigue, as well as neuropsychiatric disorders.

## Ethics Statement

Animal care and experiments were conducted in accordance with the guidelines issued by the Institutional Animal Care and Use Committee of Daejeon University (Daejeon, Republic of Korea; Approval No. DJUARB 2017-017) and the Guide for the Care and Use of Laboratory Animals published by the United States National Institutes of Health.

## Author Contributions

JL wrote the main manuscript text and conducted the experiments. WK supported the behavioral test for depression and anxiety. YJ prepared **Figure 2** and other immunofluorescence staining results. SL performed a statistical analysis. DL advised on the mechanism of NLRP3 inflammasome activation. CS supervised the preparation of manuscript and directed the final version of all contents. All authors reviewed and approved this manuscript.

## Funding

This research was supported by the Basic Science Research Program through the National Research Foundation of Korea (NRF) funded by the Oriental Medicine R&D Project, Ministry of Health & Welfare, and Republic of Korea (HI15C0112), as well as the Ministry of Education, Science and Technology (NRF-2018R1A6A1A03025221).

## Conflict of Interest Statement

The authors declare that the research was conducted in the absence of any commercial or financial relationships that could be construed as a potential conflict of interest.
