# Supplementary material for: Antidepressant-Like Activity of Myelophil via Attenuation of Microglial-Mediated Neuroinflammation in Mice Undergoing Unpredictable Chronic Mild Stress
Source: Front Pharmacol. 2019 Jun 13;10:683. doi: 10.3389/fphar.2019.00683 (PMC6585390; doi:10.3389/fphar.2019.00683)
Supplement: Supplementary file 1 [file DataSheet_1.pdf]

Supplementary Figure 1

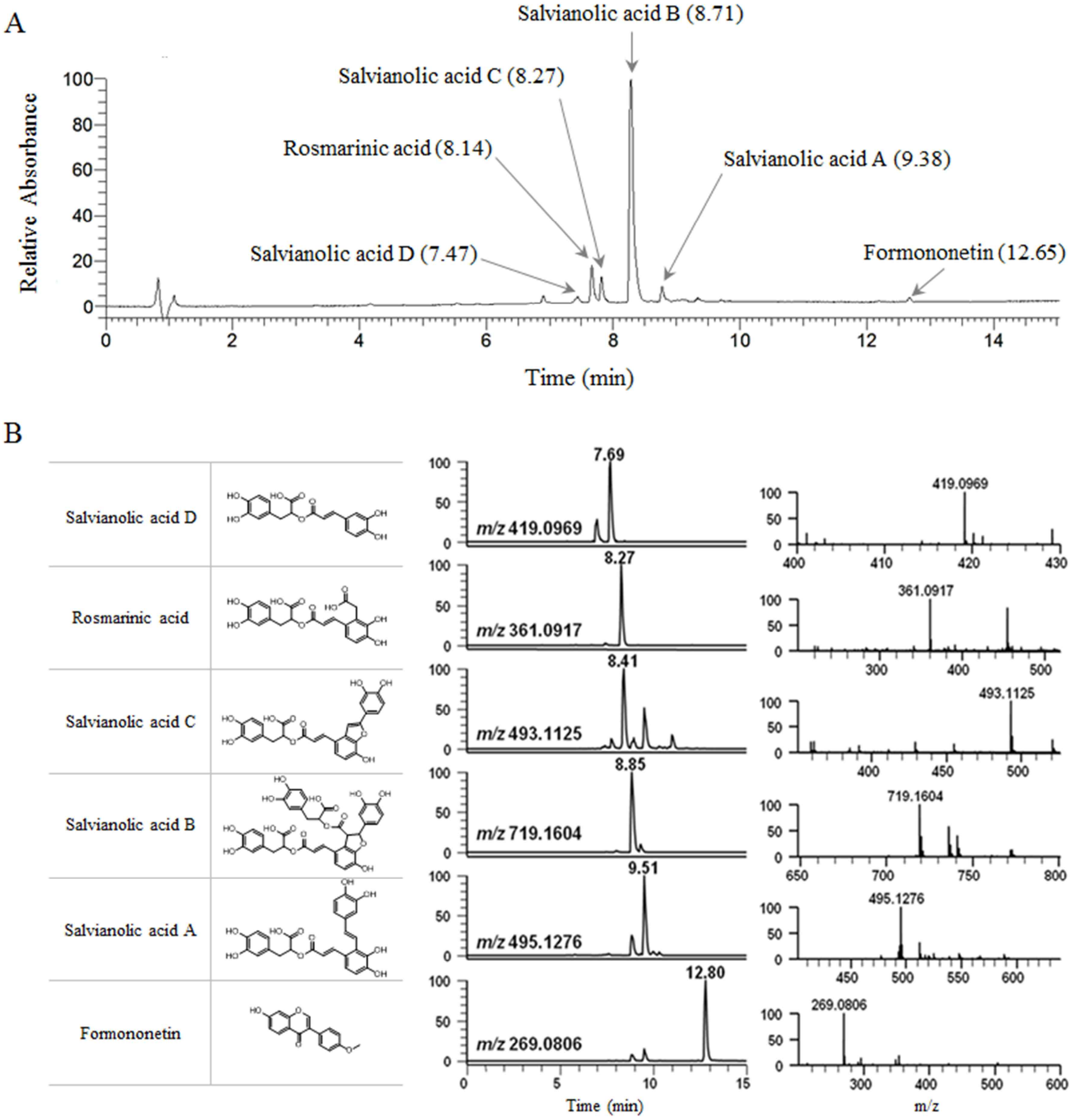

S. Figure 1. UHPLC and LC/MS chromatogram of Myelophil. Myelophil and reference compounds were subjected to UHPLC analysis (A). Structures (left), mass chromatogram (middle) and high-resolution mass spectrums (right) of 6 compound of Myelophil [salvianolic acid D (m/z 419.0969, [M+H]+); rosmarinic acid (m/z 361.0917, [M+H]+); salvianolic acid C (m/z 493.1125, [M+H]+); salvianolic acid B (m/z 719.1604, [M+H]+); salvianolic acid A (m/z 495.1276, [M+H]+); formononetin (m/z 269.0906, [M+H]+)] (B).

Supplementary Figure 2

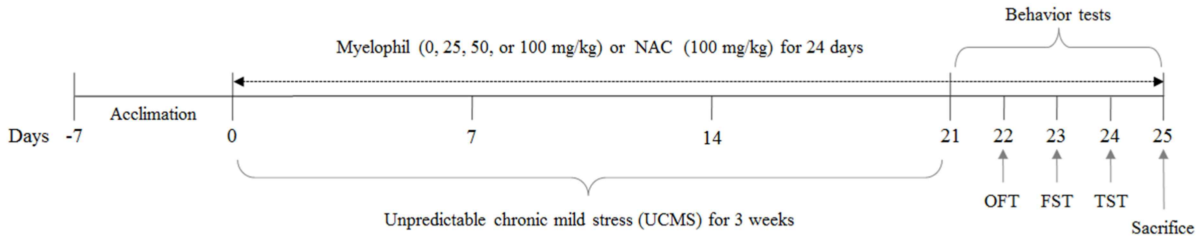

Day	Stress	Duration (condition)
1	Water deprivation	12 h (dark phase)
2	Food deprivation	12 h (dark phase)
3	Cold stress at 4°C	1 h (light phase)
4	Overnight illumination	12 h (dark phase)
5	Forced swimming	15 min (light phase)
6	Restraint	3 h (light phase)
7	Water deprivation with empty bottle	12 h (dark phase)
8	45° cage tilting	12 h (dark phase)
9	Isolation stress	24 h
10	Wet bedding	24 h
11	Cold stress at 4°C	1 h (light phase)
12	Forced swimming	15 min (light phase)
13	Overnight illumination	12 h (dark phase)
14	Wet bedding	24 h
15	Isolation stress	24 h
16	Water and food deprivation	12 h (dark phase)
17	45° cage tilting	12 h (dark phase)
18	Restraint	3 h (light phase)
19	Wet bedding	24 h
20	Overnight illumination	12 h (dark phase)
21	Water deprivation	12 h (dark phase)

S. Figure 2. Experimental scheme. Mice were subjected to UCMS procedure for 3 weeks. After the final day of stress, the depression/anxiety-like behaviors were sequentially assessed by OFT on day 22, FST on day 23, and TST on day 24. During the entire experiment period, mice were orally administered with distilled water, Myelophil or NAC, respectively. On day 25th, mice were sacrificed under CO_2 anesthesia.
